# Meaning as mentalization

**DOI:** 10.3389/fnhum.2024.1384116

**Published:** 2024-05-24

**Authors:** Bálint Forgács

**Affiliations:** ^1^Department of Experimental and Neurocognitive Psychology, Freie Universität Berlin, Berlin, Germany; ^2^Department of Cognitive Psychology, ELTE Eötvös Loránd University, Budapest, Hungary

**Keywords:** language comprehension, social cognition, semantic processing, mentalization, theory-of-mind, N400, language acquisition, pragmatics

## Abstract

The way we establish meaning has been a profound question not only in language research but in developmental science as well. The relation between linguistic form and content has been loosened up in recent pragmatic approaches to communication, showing that code-based models of language comprehension must be augmented by context-sensitive, pragmatic-inferential mechanisms to recover the speaker’s intended meaning. Language acquisition has traditionally been thought to involve building a mental lexicon and extracting syntactic rules from noisy linguistic input, while communicative-pragmatic inferences have also been argued to be indispensable. Recent research findings exploring the electrophysiological indicator of semantic processing, the N400, have raised serious questions about the traditional separation between semantic decoding and pragmatic inferential processes. The N400 appears to be sensitive to mentalization—the ability to attribute beliefs to social partners—already from its developmental onset. This finding raises the possibility that mentalization may not simply contribute to pragmatic inferences that enrich linguistic decoding processes but that the semantic system may be functioning in a fundamentally mentalistic manner. The present review first summarizes the key contributions of pragmatic models of communication to language comprehension. Then, it provides an overview of how communicative intentions are interpreted in developmental theories of communication, with a special emphasis on mentalization. Next, it discusses the sensitivity of infants to the information-transmitting potential of language, their ability to pick up its code-like features, and their capacity to track language comprehension of social partners using mentalization. In conclusion, I argue that the recovery of meaning during linguistic communication is not adequately modeled as a process of code-based semantic retrieval complemented by pragmatic inferences. Instead, the semantic system may establish meaning, as intended, during language comprehension and acquisition through mentalistic attribution of content to communicative partners.

## 1 Introduction

This study presents a new perspective on how content is transmitted during linguistic communication by proposing a novel theory of how meaning is established in the human mind. It offers an explanation for language acquisition and word learning from the perspective of mentalization—that is, the attribution of intentions, beliefs, and desires to social partners (Premack and Woodruff, 1978; Leslie, 1987). The foundation for this novel model of processing, establishing, and acquiring semantic content is based on a series of neurocognitive experiments with infants and adults. However, before introducing these studies, the broader question of the interplay between human communication, social cognition, and language comprehension will be addressed. First, I will take a closer look at the changes in thinking regarding the transmission of linguistic meaning, from the code model to pragmatic theories of communication. Next, I will explore the role of communicative intentions and how they enable language comprehension and acquisition. Then, I will discuss how social cognition is involved in utilizing language as an information transmission device and how infants employ mentalization to track the comprehension of communicative partners. Finally, I will argue that semantic processing involves the attribution of mental content through mentalization and that such mentalistic meaning-making drives and enables language acquisition and word learning.

The main claim of this study is that contrary to standard models of language comprehension, linguistic meaning does not emerge from decoding information by looking up semantic content in a mental lexicon and placing it in syntactic frames, nor from the applying rule- or relevance-based social-pragmatic inference mechanisms. While lexical retrieval and pragmatic enrichment play important roles in language processing, I argue that comprehension of meaning, as intended, fundamentally relies on attributing mental content to communicative agents as belief states. While some approaches recognize the importance of mentalization in communication, they limit its role to setting up communicative interactions (Tomasello, 2008) or reference resolution (Bloom, 2000). Both assign social cognition a key role, which is to link mental representations (of the physical world) to word forms. What distinguishes the current approach is that meaning is not identified externally in the physical world, with the help of social cognition, but internally in the mental world of communicative partners as the content of attributions of beliefs about the world.

## 2 Form and content in language

The question of how meaning is established, transmitted, and acquired is a matter of heated debate not only in linguistics but also in psychological science. In the 1950s, the dominant structuralist view on language was challenged from multiple directions. The basic assumption of this tradition was the equivalence between form and content. In contrast, the new approaches pointed out that the comprehension of linguistic meaning is only partly based on interpreting language as a code, and external factors such as communicative intentions, context, and social cognition may also play key roles.

The founder of structuralism, de Saussure, noted the arbitrariness of the connection between signifiers (form) and signified (content). His Linearity Principle promised that analyzing the sequences of signifiers would provide a systematic explanation of content (de Saussure, 1966). It also complemented Frege’s Compositionality Principle, which proposes that linguistic meaning is based on a systematic derivation of the truth-value of linguistic propositions or sentences, viewed as functions of the grammatical combination of words (Frege, 1948). Structuralism and the idea of unity between form and content remain highly influential in developmental psychology. It often serves as the hidden axiomatic assumption behind the acquisition of word-to-world mappings during word learning and the source of the expectation that language acquisition is a gradual, step-by-step process that proceeds from phonology through word learning to grammar.

The idealization of *form as content* served as the foundation for Shannon and Weaver’s information theory, which provided a mathematical formalization of communication (Shannon and Weaver, 1949). It is based on the code model, which is still the textbook model of human communication (Blackburn, 2007). This model proposes that an information source, or sender, encodes its message via a transmitter, which then sends the signal through a channel. Upon receipt, the receiver reconstructs the message by decoding it from the signal. Modeled after the telegraph, communication is formalized here as the challenge of recovering the message from signals received through a noisy transmission channel, while it takes it for granted that messages are clearly defined chunks of information and unambiguous in their content once decoded.

In the 1950s, a series of theories challenged the structuralist tradition, although not the code model itself. Wittgenstein pointed out that the relationship between form and content may be far looser than previously assumed (Wittgenstein, 1953). Chomsky proposed that linguistic meaning is, in fact, recovered from syntactic deep structures rather than from the surface forms of word sequences (Chomsky, 1957, 2015, 2017). Both of these ideas became highly influential (Pléh, 2024). Around the same time, arguments developed by two philosophers of language led to the establishment of the field of linguistic pragmatics. Austin suggested in 1955 that certain kinds of sentences function as speech acts (e.g., “thank you” or “excuse me”) that we do not evaluate based on their literal truth-value but in terms of their social force. We recognize them as not describing reality but rather bringing about some intended change in the world (Austin, 1962; Searle, 1969). Intentions were introduced as central to communication, but as Reboul and Moeschler (1998) pointed out, this approach remained unsuccessful because it tried to account for intended meaning in terms of linguistic conventions and formulae. Paul Grice was the first to suggest that communicative intentions play a key role in conveying meaning via non-linguistic inferences (Grice, 1957). He differentiated between an indicative sense of the word “meaning” (e.g., clouds may foreshadow rain) and a “meant by” sense (e.g., if someone has their head in the clouds, it expresses they live in a fantasy). Form and content are decoupled thereby: the meaning of utterances cannot be recovered by simply decoding the lexical contents of word combinations without considering speakers’ intentions in the communicative context. Grice did not elaborate on the structure or content of intentional mental states but showed that they are not only indispensable for communication but also independent of the code structure of language. When we hear the word “tiger,” we do not automatically run away assuming that the word refers to an actual tiger present in the here and now: we understand it as a communicative act, not of “indicating” but of “meaning by.”

Grice nevertheless anchored his theory in the code model when he suggested that “what was said” needs to be decoded first, based on its literal meaning and truth-value. Only afterward may one engage in the inferential mechanisms necessary to recover the implicatures, the intended meanings (Grice, 1975). Thereby, sentences are decoupled from utterances: sentences are still processed as code-based signals, but utterances become pragmatic-inferential interpretations. The precondition for these inferences is that both parties are interested in holding a conversation based on truth and trust—a concept for which Grice proposed the Cooperative Principle. Speakers may strategically violate either of four maxims (quality, quantity, relevance, and manner), the normative rules of conversations, to prompt hearers to engage in the inferential reverse engineering of the intended meaning. Here, two sets of rules exist: one for the linguistic code and another for the maxims, akin to constitutive and regulative rules, with the former establishing the framework and the latter navigating it (Black, 1962). Reboul and Moeschler (1998) argue that Grice’s theory, while underspecified to be truly cognitive, was a game changer that eventually led to the birth of experimental pragmatics (Noveck and Sperber, 2004; Noveck and Reboul, 2008; Bambini and Bara, 2010; Noveck, 2018).

Form and content are even more strongly decoupled in Relevance Theory (RT) (Sperber and Wilson, 1986; Wilson and Sperber, 2004). RT breaks with the idea that linguistic form—“what was said”—can be recovered solely based on decoding. Sperber and Wilson suggest that the language module provides an initial interpretation by constructing a logical form that serves as the premise for the inferential mechanisms interpreting utterances. However, pragmatic inferences play a role already in uncovering what was said (the explicatures), not only in what was meant (the implicatures). They also reject Grice’s Cooperative Principle and keep only one of the maxims: relevance. They suggest that relevance seeking is a general mechanism of cognition that aims to maximize effects by minimizing efforts, and it drives language comprehension as well. Instead of cooperation and normative rules, they introduce the concept of cognitive environment, which, along with the logical form, constitutes the inputs of the pragmatic inference machine. It includes physical and perceptual information as well as common knowledge, common history, and common ground. Although most examples provided by Sperber and Wilson involve mental states, on-line mentalization serves merely as an optional input for pragmatic enrichment (Mazzarella and Noveck, 2021). Alas, pragmatic-inferential mechanisms do not necessarily require cooperation or even the attribution of intentional states. Social cognition manifests in RT as ostensive communicative signals, which are attention-capturing acts that trigger relevance-seeking processes during communicative interactions.

**Figure 1:**
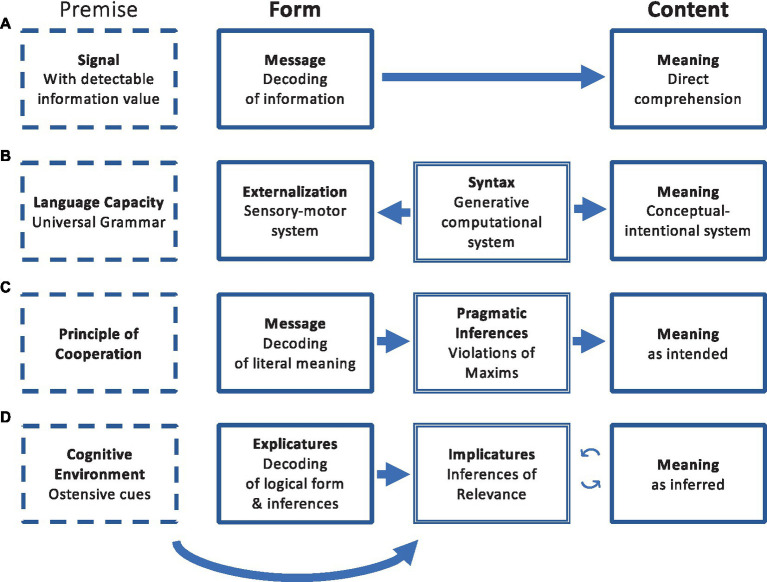
The fundamental shift in how meaning is thought to be conveyed by language, as per **(A)** the code model (Shannon and Weaver, 1949); **(B)** the generativist syntax-first approach (Chomsky, 2017); the pragmatic models of **(C)** the Gricean and Neo-Gricean (Grice, 1975); and **(D)** the Post-Gricean traditions (Sperber and Wilson, 1986). Note that “decoding” typically implies the processing of both semantic and syntactic information. Additionally, pragmatic inferences may involve some kind of social cognition (cooperation and/or ostension), but communicative intentions are thought to be derived with no attribution of mental/belief/intentional states to communicative partners.

Taken together, there has been a gradual but tectonic shift that has transformed thinking about how language conveys meaning: away from the structuralist tradition of meaning carried by linguistic forms in a code-like manner and toward meaning inferred as intended (Figure 1). While some theorists still argue that pragmatic processes enter language comprehension only when things go wrong, and until that point, language works like a code (Millikan, 2005), there now seems to be broad agreement that language is not interpreted directly through decoding. Even though the nature of inferences is hotly debated, they also all appear to be rule-based mechanisms of social cognition that do not involve the attribution of intentional or mental states to communicative partners to establish intended meaning. The cooperation-based Neo-Griceans tradition (Grice, 1975; Levinson, 2000; Horn, 2006; Goodman and Frank, 2016) argues that hearers infer the intended meaning of speakers in a serial fashion, first decoding the literal meaning of spoken language, then looking for violations (of norms/maxims or rationality/utility). The ostension-based Post-Gricean Relevance Theory camp (Sperber and Wilson, 1986; Noveck and Sperber, 2004; Noveck, 2018) suggests that hearers seek relevance while considering the linguistic input and the cognitive environment in parallel to develop implicatures. The two models agree on the central role of social cognition and communicative intentions, yet neither has put forward mechanisms that were based on mentalization, which has generated a longstanding debate about whether communication involves mentalization or not (Pléh, 2000; Bosco et al., 2018). Moreover, the term “communicative intention” is often used ambiguously in at least two different senses.

## 3 Communicative intentions in human communication

The notion of intentionality traces back to Brentano’s reintroduction of scholastic ideas. He suggested that the hallmark of psychological or mental states, such as beliefs and desires, is a kind of “aboutness”: mental events, as opposed to physical objects, are directed toward entities beyond themselves (Brentano, 2009). The question of higher-order intentionality of mental states, such as beliefs about others’ beliefs, burst into the scientific discourse in psychology with the debate about whether chimpanzees have a Theory-of-Mind (ToM) (Bennett, 1978; Dennett, 1978; Premack and Woodruff, 1978). ToM is the ability to attribute psychological states with intentionality to social partners (Jacob, 2023). In his highly influential works, Dennett proposed that humans take the “intentional stance” to predict and explain the behavior of social agents (including humans, animals, and even machines) by attributing intentional mental states, beliefs, and desires to them (Dennett, 1987).

The concept of communicative intentions has been used somewhat differently in the field of pragmatics, where it has been proposed that they may simply be recognized based on ostensive-behavioral signals without necessarily ascribing mental content to others (Sperber and Wilson, 1986). It has also been suggested that the earmark of human communication is not simply that it is intentional but rather that it is overtly intentional (Scott-Phillips, 2015). Non-human primates may be able to communicate intentionally, perhaps inferentially, but not truly ostensively (Warren and Call, 2022). Overtly intentional ostensive communicative signals call the attention of their addressee to the communicative act itself. The communicative transmission commences when an agent’s communicative intention is recognized by the addressee; the communicative intention is fulfilled when a second, informative intention is also recognized. The recognition of an informative intention is equal to comprehension and its fulfillment to believing the content. In other words, the recognition of the communicative intention opens a unique kind of communication channel, suspended from the present here and now, which allows for the transmission of information with no reference to the physical environment. In primate communication, signals may be sent informatively, even intentionally (e.g., when producing a predator alarm call to warn conspecifics), but in lack of highlighting and recognizing the communicative intention, it does not seem possible to exchange communicative signals about objects (or dangers) beyond perception. It is a species-specific feat of human communication that communicative transmissions can be about entities beyond the local physical surroundings and the present moment. Signals following the recognition of communicative intentions are suspended from perceptual reality, yet they still seem to possess a kind of aboutness akin to intentional mental states.

### 3.1 Communicative intentions in language acquisition

The critical importance of the social environment in the emergence of language was put forward by Vygotsky (1978) and Bruner (1983). In their studies, which laid the foundations for a social constructivist view on language, the social world is discussed as a special kind of environment, distinct from the physical world. It is an indispensable yet external context for learning and development, not an internal matter of mind and cognition (Pléh, 2024). Additionally, their basic assumption, like many others’, has been that communication requires a code-based signaling system, that is, language. The idea that human communication is built on social cognition, as it is not mere information transmission, reverses the above order: communication may need to precede language acquisition. There are two dominant views regarding the developmental origins of human-specific communication and the role social cognition plays in it. Tomasello (2008), by and large, follows the (Neo-) Gricean tradition in emphasizing cooperation as the basis of communication and language in his joint attention framework; Gergely and Csibra’s Natural Pedagogy theory fits well with RT and the Post-Gricean perspective when proposing that ostensive cues play a central role in communicative interactions even in preverbal infants (Csibra and Gergely, 2009; Gergely and Csibra, 2013).

#### 3.1.1 Tomasello’s shared intentionality infrastructure

To “break into the code” of language (as Tomasello puts it) to decipher its syntactic and semantic structures, children need to be able to communicate in some way from the outset. Tomasello argues that this initial form of communication is founded upon a dedicated “cognitive infrastructure” of “shared intentionality.” Pointing and pantomiming are not based on preestablished conventional codes, yet infants can use them communicatively. These preverbal communicative acts presuppose sensitivity to cooperation (missing in our closest primate relatives) and are based on shared intentionality, an understanding that *we*, as social partners, may intend things together. Having a joint goal is not merely two agents having the same goal simultaneously, like aiming to reach the same destination, but like walking together. Humans’ propensity for cooperation to pursue joint goals provides the context in which communicative acts such as pointing or pantomiming acquire a shared meaning. This joint context, the common ground (Clark, 1996), is indispensable for interpreting actions as communicative gestures. It is established by what Tomasello calls “joint attention,” when participants are aware that they simultaneously attend to the same object. Tomasello specifies three basic human communicative intentions enabled by the above cooperative infrastructure: requesting, informing, and sharing. To fulfill such intentions, participants need to reason not simply practically (i.e., rationally) but cooperatively, relying on their partners’ helping attitude.

This kind of human cooperation that emerges around 9–12 months of age in human ontogeny, Tomasello argues, requires “recursive intention-reading and mind-reading abilities.” Phylogenetically, these abilities are supposed to originate from the ability to establish joint goals, which led to the emergence of joint attention and eventually enabled the establishing of common ground. At the same time, Tomasello also argues that children under 4 years of age possess only rudimentary mentalization abilities akin to the ToM of great apes (Tomasello, 2018)—which is nevertheless still sufficient to support the recursive mind-reading necessary for the earliest forms of human communication. Shared intentionality is thus a feat of cooperation, not of ToM. Others have also argued that some level of mentalization may be indispensable for the attribution of goals (Csibra and Gergely, 2007) or attention (Elekes and Király, 2021), but it is not clear how sophisticated these ToM representations need to be to support “shared goals” and “joint attention.” In Tomasello’s view, recursive mindreading is present already in 9-month-olds, but it does not seem to play a significant role in the transmission of information, as the shared intentionality infrastructure and attention-checking may be sufficient to sustain the earliest forms of human communicative interactions.

Such a framing, while placing cooperation at the center, turns Grice’s account upside down. Instead of first decoding content and then enriching it with cooperation-based pragmatic inferences, it is now sensitivity to cooperation that allows for information transmission. It is the common ground that allows for interpreting, in Tomasello’s terms, the “natural” signals of pointing and pantomiming and eventually the “conventional” linguistic signals. Language, however, is just a ritualization of communicative interactions, not qualitatively different from non-conventional forms of cooperative communication. Tomasello points out that the first words appear in and emerge from cooperative routines (Bruner, 1983) when infants understand the intentional structure of the shared goal and begin to reason cooperatively (Tomasello, 2008).

#### 3.1.2 The ostensive signals of Natural Pedagogy

Gergely and Csibra’s Natural Pedagogy theory sidesteps the issue of mindreading when it proposes that perceptually identifiable ostensive-behavioral signals may account for human-specific communicative interactions (Csibra and Gergely, 2009). In contrast to RT, the primary role of ostensive signals is not to support inferential communication but to enable cultural learning (Gergely and Csibra, 2005). They allow learners to identify and recognize the communicative, pedagogical intention of knowledgeable partners to transmit culturally relevant knowledge (Gergely and Csibra, 2013). Here, ostensive signals do not simply function to capture attention, as in RT; rather, particular attention-grabbing signals are employed to induce cultural learning because they are ostensive (Gergely, 2010). There is evidence for at least three, perhaps innately specified ostensive signals that may be recognized as indicating communicative intentions already around 4–5 months of age (Grossmann et al., 2007; Parise et al., 2008; Parise and Csibra, 2013): eye-contact, contingent (turn-taking) reactivity, and infant-directed speech. While code-based signals, in general, are poor means of information transmission without pragmatic inferences, ostensive signals can be utilized in a code-like manner to induce the recognition of communicative intentions (Csibra, 2010).

Natural Pedagogy puts forward a mechanism for establishing common ground not based on cooperation but on evolved, trust-based procedures that pick out certain behavioral cues as ostensive signals. These signals do not merely activate attentional resources but also initiate species-unique cultural learning strategies. When addressed ostensively, infants assume that the information transmitted is generalizable, socially and culturally shared, and constitutes normative knowledge (Gergely et al., 2002; Yoon et al., 2008; Futó et al., 2010; Hernik and Csibra, 2015). One outstanding example of cultural learning in natural pedagogical situations is the acquisition of word labels, which are indeed socially shared and mostly refer to kinds. Taken together, Natural Pedagogy is an evolved system that serves social knowledge transmission underlying cultural learning just as well as human communication.

### 3.2 Mentalization and communicative intentions?

Although the Neo-Gricean and the Post-Gricean models are not mutually exclusive, they emphasize different aspects of communicative interactions and assume different sufficiency and necessity conditions for establishing common ground. Both models are primarily interested in explaining how to identify communicative intentions, with less emphasis on how information is actually transmitted (i.e., the informative intention). While communicative intentions may be recognized either through a code-like signaling system (ostension) or through the motivation for cooperation (joint attention), neither model argues for the necessity of mentalization, even though both involve attending to a social partner and identifying the partner’s focus of attention as a referent.

Another person’s attention or goal (i.e., the referent of a communicative interaction), however, cannot be but an attribution of attention (Elekes and Király, 2021) or of a goal (Csibra and Gergely, 2007). It may be argued that recognizing the attention or the goals of social partners relies on some sort of non-mentalistic mechanism, for example, on teleology (Gergely and Csibra, 2003). However, teleology does not seem to suffice to explain communication non-mentalistically because in communicative interactions, referent objects are not the goals of agents but the goals of the communication itself. Moreover, information transmission takes place only after minds establish common ground through joint attention and/or ostensive signals. Yet, minds never literally “join”; thus, common ground can emerge only in the minds of the two interlocutors separately. The two parties may mutually assume that the other has an identical belief about the state of affairs as themselves, but this can only be an ascription of a mental state to the other party. The point is that the machineries proposed for setting up communicative interactions by identifying communicative intentions are fundamentally attention-directing and attention-checking systems. These systems are aimed at identifying what the partner is calling the infant’s attention to, which is the partner’s own focus of attention, to establish that both parties have the same referent in mind. Perhaps due to the ambiguity of the term “communicative intentions,” a considerable group of researchers seems to believe it is obvious that language, like communication, involves mentalization, while another large group appears to hold it is rather obvious that neither communication nor language does.

### 3.3 Timing and difficulty of inferring communicative intentions?

There is another intriguing contradiction regarding pragmatic inferences, as highlighted by Bohn and Frank (2019). There is a long line of research arguing that infants reason skillfully about intentions already during language acquisition (Nelson, 1973; Bates, 1976; Bloom, 2000; Tomasello, 2003). Another line of research reports the difficulties children experience in deriving pragmatic inferences to interpret intended meanings (Papafragou and Musolino, 2003; Huang and Snedeker, 2009). Bohn and Frank propose to resolve the contradiction by defining communication as social cognition and reasoning about the goals of communicative partners. The Rational Speech Act framework suggests that pragmatic reasoning integrates all the elements of inferential communication, which are in place early on and foster the gradual development of language comprehension (Bohn and Frank, 2019).

The apparent contradiction may stem from the ambiguity of the term “communicative intentions.” It may refer to the social-cognitive inferential mechanisms employed to identify the *intent to communicate*, which may be present from early infancy onward on the one hand. It may also refer to the inferential mechanisms applied to the transmitted information content to recover *meaning as intended*, which may be challenging even for kids, on the other. While the former is the utilization of pragmatic-inferential mechanisms to set up a communicative infrastructure for the upcoming information (*cf.* Tomasello), the latter involves enriching the already available information with pragmatic inferences (*cf.* Grice).

The above two kinds of ambiguity surrounding the term “communicative intention”—(1) whether it is mentalistic or not and (2) whether interpreting it appropriately is easy (already in infancy) or difficult (even in late childhood) —seem to be orthogonal (Table 1). Some argue that inferring communicative intentions is essential for word learning, is available already in infancy, and involves mentalization (Bloom, 2000; Papafragou, 2002; Tomasello, 2003; Thompson, 2014). Others assume that word learning is based on inferential mechanisms, but it does not require mentalization (Sperber and Wilson, 1986; Csibra and Gergely, 2009; Bohn and Frank, 2019). Some show how non-mentalistic pragmatic inferences are challenging for kids when interpreting, for example, scalar inferences (Papafragou and Musolino, 2003; Huang and Snedeker, 2009; Noveck, 2018), contrastive inferences (Kronmüller et al., 2014), or logical terms (Noveck and Chevaux, 2002; Pouscoulous and Noveck, 2009). Finally, the position that pragmatic inferences develop slowly but co-develop with and involve mentalization has also been put forward (Rubio-Fernandez, 2021). The debate boils down to two key questions: (1) can communicative intentions be truly non-mentalistic (i.e., inferred based on code-like signals or regulative rules) and (2) what level of mentalization may be available in infancy (i.e., to what extent language acquisition may or should be tied to it)? Both of these questions are going to be addressed later on.

**Table 1:** The various interpretations and uses of the term “communicative intentions” in the literature.

Communicative intentions	Mentalistic	Non-mentalistic
Easy to infer	Bloom (2000), Papafragou (2002), Thompson (2014), and Tomasello (2003)	Bohn and Frank (2019), Csibra and Gergely (2009), and Sperber and Wilson (1986)
Difficult to infer	Rubio-Fernandez (2021)	Huang and Snedeker (2009), Noveck (2018), Noveck and Chevaux (2002), Papafragou and Musolino (2003), and Pouscoulous and Noveck (2009)

Taken together, there seems to be an agreement that social cognition, in the form of pragmatic inferences, plays a key role both in setting up communicative interactions and in deriving the implied meaning of utterances behind words and sentences. The first mechanism appears to precede the transmission of the linguistic code (i.e., setting up a communicative interaction, either via cooperation and joint attention or via ostensive communicative signals), while the second one follows it (i.e., enriching it inferentially to interpret it, either via checking for violations of maxims in a cooperative framework or via carrying out a relevance-based calculus). The first one seems to pertain to “communicative intentions” in the narrow sense: a scaffold of informative intentions. The second one uses the term in the broad sense: inferring meaning as intended. It may be argued that the latter actually concerns informative intentions, but this is not entirely clear from the literature. “Intended meaning” is typically referred to as what was intended to be conveyed (i.e., communicated), not what one was intended to be informed of. The term “information” may be the culprit here: it could mean either the content (utterance) or the form (sentence)—perhaps due to the remarkable influence of the code model. Notably, the question of how social cognition modulates the information content, specifically the link between linguistic form and semantic content, seems to have gathered limited attention. Yet, the linguistic signal is the most variable and rich source of input entering pragmatic inferential mechanisms.

## 4 Language as an information transmission device

While human communication may be viewed as a form of social cognition based on pragmatic inferences that enable language acquisition and comprehension (Tomasello, 2008; Bohn and Frank, 2019), it may also be viewed as a tool for information transmission (Shannon and Weaver, 1949; Tauzin and Gergely, 2018, 2019). Of course, these two views are not mutually exclusive, and, in some sense, they represent two sides of the same coin. Nevertheless, they still represent fundamentally different views on the role language plays in communication. In the former view, meaning emerges primarily from the common ground and the structure of the social interaction (Clark, 1996; Tomasello, 2008; Bohn and Frank, 2019), while in the latter, it arises from the properties and the variability of the signal, perhaps in interaction with mental states (Tauzin and Gergely, 2018).

The signal variability approach gains particular relevance due to the richness of the contents that may be transmitted in spoken language, from storytelling to discussions of shared memories. The communicative goal of verbal interactions is often far beyond the social situation and maybe more intricate than the basic intentions of requesting, informing, or sharing (Tomasello, 2008). Narrative stories rely heavily on information transmission to set up the communicational situation itself. It follows that the information content—the intended meaning—may be at least partly recoverable from the mental state of communicative partners rather than solely from the common ground or the situational features of the cognitive environment. Linguistic forms may have reached the unmatched level of signal complexity precisely because they may be more about what the partner could have in mind and less about the social interactions of relatively limited complexity, especially those in which non-human and young human apes typically engage.

### 4.1 Sensitivity to the code-like features of language in neonates

From an information transmission point of view, it may not be surprising that language, as a stimulus class, enjoys a special status in human ontogeny. Well before birth, prenatal humans begin to pick up the prosodic properties of their native tongue (Abboub et al., 2016), and right at birth, they are sensitive to a range of physical-acoustic and phonological features of language (Werker and Tees, 1999). Newborns prefer speech to matched non-speech, forward-going speech to backward speech, their mother’s voice to other female voices, and their native language to unfamiliar languages; they can also differentiate between languages based on rhythmic properties even if they have never heard them before and can detect word boundaries, discriminate lexical stress, and even distinguish function words from content words based on acoustic characteristics (reviewed by Gervain and Mehler, 2010).

During language acquisition, infants use several of these acoustic properties, including rhythm (Goswami, 2022) and statistical distributional patterns (Kujala et al., 2023), to “break into the code” of language by approaching it from its information-transmitting potential. The acoustic-phonological-prosodic properties of language lend themselves readily to being deciphered as a code. First, the set of sounds the human vocal tract can produce is limited, making it computationally manageable. Second, phonology is not only governed by rules but also carries information about higher-level syntactic operations, both of which involve code-based computational structures. Even newborns rely on prosody and statistical learning of transitional probabilities to identify word boundaries (Fló et al., 2019). To segment the continuous speech stream into potentially meaningful units, they also employ various dedicated mechanisms to learn about the segments themselves (Fló et al., 2022). They also utilize innate pattern recognition mechanisms to pick up repetition structures (i.e., pseudowords with ABB structure, e.g., “mi-zu-zu,” as opposed to ABC random structures, e.g., “mi-zu-ka”) (Gervain et al., 2008). The repetitions may be engaging for them because they reveal a rule that may indicate syntactic structures, thereby providing a better opportunity to learn (about) language. Such sensitivity to rule-like structures extends to musical tones as well; however, only pseudowords, not tones, activate the left inferior frontal regions (Nallet et al., 2023). These regions include Broca’s area, which is responsible for processing the structural properties of language in adults (Musso et al., 2003; Liakakis et al., 2011; Friederici, 2012) and responds to language at birth (Peña et al., 2003; Perani et al., 2011). Newborns detect not only repetition structures but also their sequential position (ABB vs. AAB), and these two properties seem to be the two fundamental building blocks of any code-based system (Gervain et al., 2012). These findings strongly suggest that language is a unique signal for humans, engaging dedicated mechanisms, from statistical learning to pattern recognition, to identify word-like units and grammar-like rules right from birth. Newborns appear to be very well equipped to unpack the structural properties of the code system humans use to transmit information.

### 4.2 Communicative self-referentiality in the speech signal

In the process of acquiring linguistic meaning, the only communicative cue that has been suggested to signal communicative intentions and is linguistic in nature is infant-directed speech (IDS) (Csibra and Gergely, 2009; Gergely, 2010); also called “motherese,” its characteristic prosodic pattern includes higher and broader pitch, greater amplitude variation, and slower speed than typical adult speech (Csibra, 2010). Although there seems to be some cultural variation (Cristia, 2023), sensitivity to IDS appears to be innate, present at birth (Cooper and Aslin, 1990), and universal (Fernald, 1992). The perceptual features of motherese appear to open the gateway toward the content of speech: IDS may simultaneously carry information about communicative and informative intentions. In fact, Sperber and Wilson (1986) consider language to be an ostensive cue in and of itself. Every communicative act carries its own relevance by definition, and a linguistic utterance is clearly communicative—at least for adults. Infants may exploit the prosodic layer of IDS for communicative intentions to gain access to its contents (i.e., informative intentions). IDS modulates electrophysiological responses to faces in 4-month-olds, perhaps because it generates communicative expectations (Sirri et al., 2020). It is interpreted as an ostensive cue by 5–6-month-olds, just as eye-gaze (Senju and Csibra, 2008; Parise and Csibra, 2013; Lloyd-Fox et al., 2015). It also facilitates 7-month-olds’ cortical tracking of speech (Kalashnikova et al., 2018). IDS appears to serve as a self-referential linguistic inroad toward linguistic meaning as it induces a communicative interpretation of the linguistic code. As an ostensive signal, it creates an expectation that incoming information refers to kinds and not individuals (Gergely and Csibra, 2013), which clearly aids word learning since, with the exception of proper names, words refer to categories.

### 4.3 Recognizing the communicative function of language

Infants also appear to realize early on that language may carry information. In a series of remarkable experiments, Vouloumanos and colleagues (Martin et al., 2012; Vouloumanos et al., 2012; Vouloumanos, 2018) showed that infants expect speech—but not coughing or humming—to transmit information about intentions (i.e., objects preferences). Even 6-month-olds showed this expectation even when they had no chance to understand the transmission because it was non-sensical or foreign to them (Vouloumanos et al., 2014). Eleven-month-olds may prefer to interact with native speakers because they expect them to share information (Begus et al., 2016). Intriguingly, signal properties may modulate such expectations.

Using the so-called Flatfish paradigm, Tauzin and Gergely demonstrated that 10.5-month-old infants identify beeping entities as agents with preferences, but only if they exchange varying tone signals—not if they parrot each other using identical signals (Tauzin and Gergely, 2019). The authors argue that, from an information theoretical perspective, there are two elementary building blocks of communication: (1) there are two agents taking turns exchanging signals, and (2) the signals vary in an optimal way, with a high-but-imperfect level of contingency. The exchanged signals need to be similar enough to form a correspondence yet different enough to carry added information value (Tauzin and Gergely, 2021). At 13 months of age, infants assume a flatfish to update its falsely held belief about the location of an object only after an optimally variable signal exchange with another flatfish (Tauzin and Gergely, 2018). Communication, as information transmission, may directly modify mental state attribution through signal variation, irrespective of social-pragmatic inferences.

According to a recent study, humans may be sensitive to the information transmission value of language already at birth. When presented with grammatically structured ABB pseudowords, but now as an exchange between a female and a male voice, newborns showed increased activity near Broca’s area when the pseudowords were different tokens (female: kamumu; male: dekiki) compared to when they were identical (male: bulili; female: bulili) (Forgács et al., 2022a). These findings demonstrate, first, that neonates can identify the possibility of information transmission in communicative interactions, even when they may have no idea about its contents. Notably, they can do so even when they are not participants in the interaction and without relying on social cognition. If turn-taking were interesting in and of itself, there should have been no difference between the identical and variable signal exchanges. Second, the activation of Broca’s area suggests that it is the language-processing region of the brain that responds to the possibility of information transmission. Processing the potential for information transmission may be a core feature of human language.

The sensitivity to information value is independent of any particular semantic content or the structure of social interactions. In contrast, it is markedly missing from pragmatic models of human communication (Sperber and Wilson, 1986; Grice, 1989; Tomasello, 2008; Goodman and Frank, 2016). These models assume that information is transmitted as a code and then enriched and inferentially unpacked by social cognition. Yet, humans seem to be sensitive to information transmission even without knowing any code. Humans at birth appear to possess a structured representational template of an informative intention embedded within a communicative intention, allowing them to identify communicative intentions even when the embedded representational slot for the informative intention remains empty and even in the absence of social cues directed toward them. Of course, the above study did not provide direct evidence of a second-order representation or the recognition of a communicative intention or the relationship between the two; thus, these interpretations remain just as hypothetical as they are for infants (Csibra, 2010). Nevertheless, the underlying information estimation mechanism may be a third route for identifying communicative intentions alongside joint attention and ostension. Note that the ostensive cue of turn-taking is based on tracking proximal contingencies in interactions infants are part of, while the information estimation route capitalizes on distal contingencies.

Newborns’ sensitivity to information structure implies that humans may assume the existence of a code with content that can be sent and received, which may be just as important to language acquisition and processing as syntax and social cognition. This possibility is in sharp contrast with both Chomsky’s and Tomasello’s proposals. Chomsky suggests that syntax is the core feature of cognition in the form of recursion (Chomsky, 1965; Hauser et al., 2002) or merge (Berwick and Chomsky, 2011). However, its externalization, spoken language and communication are of no particular interest. Tomasello’s (2008) work implies that information can be transmitted communicatively only once the cognitive infrastructure for shared intentionality emerges. The notion that information transmission may be identified based on signal variability hints that humans may be able to enter the suspended space of human communication beyond the here and now, right from the very beginning of life—and language acquisition. Information may be identified without awareness of any form, content, or social context. But how may the actual meaning of language be figured out?

## 5 The emergence of linguistic meaning

Humans arrive in our world with an impressive cognitive arsenal to acquire language. They are well-prepared to unpack the code-like features and structures of phonology and syntax. They are endowed with inferential tools of social cognition to engage in human communication and have some understanding of information transmission to recognize communicative intentions. For information content not only to be identified but also to be learned, that is, for linguistic forms to be connected to conceptual knowledge, meaning needs to emerge within communicative interactions.

Social cognition has been proposed to play an important role in communication, even in the animal kingdom (Fitch et al., 2010), and to aid language acquisition throughout human development. There is a rich literature on how gaze-following (Brooks and Meltzoff, 2008, 2013), indicative of attention (Baldwin and Markman, 1989; Baldwin, 1993), or perspective-taking (Nadig and Sedivy, 2002; Nilsen and Graham, 2009; Khu et al., 2018) enables reference resolution; on how infants are able to exploit ostensive eye-gaze and pointing (Behne et al., 2005), iconic gestures (Bohn et al., 2019), and ostensive cues in communicative situations (Egyed et al., 2013); or on how the ability to use gaze, pointing, and other communicative gestures fosters later referential language production (Carpenter et al., 1998). The list is long, with excellent reviews (Clark and Amaral, 2010; Bohn and Frank, 2019) and meta-analyses available (Lewis et al., 2016; Bergmann et al., 2018). More radical forms of pragmatic-constructivist theories of language acquisition suggest that instead of word-referent mappings (Bloom, 2000), meaning is based on usage (Tomasello, 2003) or that usage may even start without meaning (Nelson, 2009). The broad agreement in developmental science is that social cognition, in the form of pragmatic inferences, plays a fundamental role in language acquisition.

In this social-pragmatic line of research, meaning is traced back to communicative intentions but is inferred from the social context, not attributed to the communicative partner. Communicative intentions are supposed to be formed by *assuming* goals or attention, not by *attributing* intentionality. Moreover, most, if not all, pragmatic inference mechanisms involving gaze-following, pointing, perspective taking, or gestures mainly, if not exclusively, target reference resolution—the content of communicative exchanges. Reference resolution is the point at which the promiscuously used term “communicative intention” switches from its sense of “intending to behave communicatively” to its other sense of “intending to express a particular meaning.” Meaning, as identified by attention and goal tracking mechanisms, is assumed to be linked to a referent in the outside world in the form of an object (nouns), an action (verbs), or a property (adjectives). The role of social cognition is to narrow the communicative interaction to the appropriate property of the physical environment, but it does not have much to do with meaning *per se*. While it is controversial whether communicative intentions involve mentalization, at least in the strict sense of attributing false beliefs, whether informative intentions—i.e., referential information transmission—may have anything to do with mindreading is not even considered.

Moreover, it is not entirely clear when and where meaning, as comprehension, emerges during communication. In the Neo-Gricean tradition, inferences are applied only after a literal meaning is decoded. The informative intention is treated as the code and the communicative intention as the pragmatic inference; thus, meaning emerges after the second step. In the Post-Gricean approach, once communicative intentions are recognized, inferences are employed to develop both explicatures and implicatures. It is the recognition of the informative intention that yields an accurate interpretation of meaning as inferred. Thereby, communicative intentions could contribute to meaning in two ways. Either in the broad sense by inferring the intended meaning (i.e., deriving implicatures) at a late stage. Or in the narrow sense, intending to communicate at an early stage. In the latter case, however, meaning (i.e., implicature) is computed at the level of embedded informative intentions.

Whether any of the pragmatic inferences employed to derive meaning involve mentalization remains unresolved. First, for the Neo-Griceans, ToM may contribute to communication either before language processing proper (*cf.* Tomasello’s shared intentionality) or after decoding, during the pragmatic inference stage (*cf.* Grice’s enrichment). Even though the Rational Speech Act theory suggests a fully integrated mechanism (Bohn et al., 2021), it is still based on literal meaning, which presupposes encapsulated decoding (Goodman and Frank, 2016; Bohn and Frank, 2019). It also remains uncommitted as to whether mentalization contributes to the integrated pragmatic inferences that yield meaning. For the Post-Griceans, mentalization is an optional input, along the logical frame, for pragmatic inferences (Mazzarella and Noveck, 2021). The initial decoding is thus sufficient for identifying encyclopedic entries but insufficient to convey meaning. Nonetheless, since mentalization is optional, it does not seem necessary for meaning. Taken together, the question of whether there can be word learning without the attribution of mental states remains unanswered. Pragmatic theories argue for the decisive role of pragmatic inferences, either before (to identify the intention to communicate) or after words are decoded (to reason about their possible content), but they downplay or omit the role of mental states in the comprehension of meaning, despite building their arguments on intentions, communicative in nature.

### 5.1 Word learning: meaning as the merger form and content?

The idea that meaning emerges by establishing word-to-world mappings (Waxman and Lidz, 2007) via linking objects to sounds can be traced back at least to John Locke (Locke, 1975). In fact, it may be a unique feat of our species that a single system, rather than two separate ones, handles both conceptual representations and communication (Miller, 1990). The way these connections are established is still debated, however. The classical view of associations (Hume, 1978; Sloutsky et al., 2017), a form of statistical learning (Smith and Yu, 2008), has been seriously questioned on the grounds of social-pragmatic cognition (Tomasello, 2003; Bohn and Frank, 2019) and by placing intentions at the center stage (Macnamara, 1972; Bloom, 2000).

One outstanding challenge in explaining word learning is the question of referentiality. Referentiality is the idea that words single out and point to things in the world. However, they do so not at the level of individuals—and based on associations—but at the level of kinds (Waxman and Gelman, 2009). This definition is a minimalist one because proper names pick out individuals, but it suffices for most words. According to a series of well-crafted studies, when objects are labeled consistently, with pseudowords rather than tones, 3-month-olds form categories based on sets of objects and generalize membership to previously unseen novel members (Perszyk and Waxman, 2018). On the other side of the same coin, words refer also in the sense that they pick out objects. It has been shown that 4-month-olds follow the gaze direction of an actor faster to locate an object if the actor utters a pseudoword beforehand—backward speech, no vocalization, or looking at the infant instead of the side of the screen where the object is to appear do not do the trick (Marno et al., 2015). These findings show that very young infants can link linguistic signals to conceptual categories and expect these signals to indicate objects.

The first word infants seem to grasp is their own name, at least by 5 months of age (Grossmann et al., 2010; Parise et al., 2010). They do not take long to have at least some understanding of at least some—food-related and body-part—words by 6 months of age (Bergelson and Swingley, 2012, 2015; Tincoff and Jusczyk, 2012). Even these first words are organized in a semantically structured manner (Bergelson and Aslin, 2017), although word frequency and cross-linguistic differences may play a role here (Kartushina and Mayor, 2019; Steil et al., 2021). These findings refuted the long-held idea that during the first year of life, infants primarily learn the phonology of their native language(s) and that word learning proper begins only around their first birthday (Bloom, 2000; Kuhl, 2011).

Word comprehension undergoes qualitative changes during the first year, nevertheless. An electrophysiological indicator of semantic processing, the so-called N400 event-related potential (ERP) (Kutas and Hillyard, 1983; Kutas and Federmeier, 2011), can be elicited in infants by mislabeling objects (Friedrich and Friederici, 2004; Parise and Csibra, 2012). It appears as early as 6 months of age but only during the encoding phase of novel object-label pairings; a day later, infants show only a so-called N200-N500 phonological familiarity effect (Friedrich and Friederici, 2011). These results reveal that word forms are processed and semantic memory structures are in place but function at a limited capacity in 6-month-olds. Even at 9 months of age, the semantic system requires some support to produce an N400, such as words being produced by the infants’ caregiver instead of by an experimenter (Parise and Csibra, 2012) or infants being familiarized with word labels in the lab (Junge et al., 2012). Only the top third high word producers of 12-month-olds exhibit the N400, and it can be reliably evoked only in 14-month-olds (Friedrich and Friederici, 2005, 2008; Forgács et al., 2019). A turning point in word learning at 14 months of age is underscored by the dramatic increase in infants’ performance in Bergelson and Swingley’s (2012) data as well. Werker and colleagues also demonstrated that only 14-month-olds, but not 12-month-olds, can link objects with labels during habituation training (Werker et al., 1998). A boost in the acquisition of abstract words has also been reported in this age group (Bergelson and Swingley, 2013), as well as a more sophisticated understanding of common ground (Moll et al., 2008). These shifts occur right before the onset of the supposed vocabulary spurt, an intense, albeit debated, expansion of the mental lexicon (Bloom, 2000; McMurray, 2007).

To expand their vocabulary, kids are thought to employ a number of dedicated learning strategies—not simply general inductive mechanisms. They rely on word learning constraints (Markman, 1990), such as the whole-object assumption, the taxonomic assumption—from 18 months of age (Markman and Hutchinson, 1984)—and the mutual exclusivity assumption—from as early as 12 months of age (Pomiechowska et al., 2021). They also utilize semantic (Pinker, 1984) and syntactic bootstrapping mechanisms (Brown, 1958; Gleitman, 1990), whereby they infer the meaning of words based on the meaning of the surrounding words in the former and by their syntactic role in sentences in the latter case. Taken together, the semantic system, which is thought to store the meaning of words, seems to be operational from 6 months of age and fully functional by 14 months of age. Word learning is thought to be aided by social cognition, which is thought to be external to the semantic system and pertaining mostly to pragmatic interpretative mechanisms.

### 5.2 Mentalization in the interpretation of the meaning of language

Just as with the diverse use of the term “communicative intentions,” there is a continuum among researchers who advocate for the role of mentalization in acquiring the meaning of words and those arguing against it. Those who believe that mentalization is crucial on the road toward linguistic meaning—beyond the recognition of communicative intentions—mostly aim to account for referent resolution (Bloom, 2000; Tomasello, 2008). Tomasello’s (2008) line of reasoning practically seeks to resolve referential ambiguity: recursive mind reading is necessary for appreciating shared goals, which creates joint attention, giving rise to common ground, which in turn allows for identifying the content of pointing, pantomiming, or words. Even those who do not explicitly argue for mentalization in referent resolution rely on some form of attention-guiding mechanism (Baldwin, 1991). Ostensive cues play a very similar role when they serve to establish a cultural learning interaction and, thereby, a unique interpretative context by guiding attention toward objects (for nouns), actions (for verbs), or functions/properties (for adjectives) (Csibra and Gergely, 2009). However, attention tracking may not be a good substitute for mentalization, as it still requires an attribution (Elekes and Király, 2021). One important motivation for leaving out mentalization from language acquisition has been uncertainty about whether ToM is available before 4 years of age. Pragmatics may have seemed a safe place to introduce mentalization in language acquisition because it fitted well with an unspoken, linear developmental order and the sequential conceptions of online language processing inherited from the serial comprehension models of Grice and Chomsky.

### 5.3 Developmental psychology’s debate: language for ToM or ToM for language?

When the question of ToM was first raised in cognitive science (Premack and Woodruff, 1978), it soon became a tool to explain autism spectrum disorder (ASD) (Baron-Cohen et al., 1985; Frith and Happé, 1994). Autism had previously been treated mainly as a language deficit, but the new argument was that ASD children are unable to learn to use language in a socially appropriate manner because of a lack of a well-functioning ToM module and concomitant reduction in social motivations that curtail the necessary linguistic input. In an interesting twist, this idea was reversed while researchers scrambled to explain the classic explicit ToM tasks such as the Sally-Anne or Maxi task (Wimmer and Perner, 1983). The argument shifted to the idea that it was language development that enabled ToM (de Villiers and de Villiers, 2000), although the possibility of bidirectional influences was also offered (de Villiers, 2007). Some proposed the necessity of semantic development: as conceptual enrichment unfolds hand-in-hand with word learning (Gopnik and Meltzoff, 1998), ToM becomes available through learning mental words such as “think” or “believe” (Olson, 1988; Brooks and Meltzoff, 2015). Others emphasized that the emergence of ToM depends on grammatical structures (de Villiers, 1998; de Villiers and Pyers, 2002; Hale and Tager-Flusberg, 2003). Paralleling the finding that the acquisition of mental words is aided by complement clauses (“thinking or believing *that*”) (Papafragou et al., 2007), mental state attribution is made possible by learning the syntactic structure for embedding propositions into propositions, in a meta-representational format (“Maxi thinks that »the chocolate is in the cupboard«”). Again, others have argued for the role of pragmatics (Harris et al., 2005; Frank, 2018; Rubio-Fernandez, 2021). A meta-analysis found that language indeed exerts a considerable influence on ToM: syntax and semantics, alongside receptive vocabulary size, memory for complements, and general language ability, were all positively associated with it (Milligan et al., 2007).

This direction of thinking has taken for granted, however, that ToM becomes available only once kids are able to pass explicit ToM tasks around 4 years of age (Wimmer and Perner, 1983). In such paradigms—the Maxi, the Sally-Anne, or the Smarties task (Perner et al., 1987)—children are explicitly asked about the mental contents of social partners (e.g., “What does Sally think, where are her marbles?”). Perhaps it is no wonder that language competence and ToM abilities have consistently been found to be interrelated.

When it emerged that preverbal infants exhibited ToM abilities (Scott and Baillargeon, 2017) as early as 6–8 months of age (Kovács et al., 2010; Southgate and Vernetti, 2014; Kampis et al., 2015), the idea that various language abilities lay the foundations for ToM was seriously challenged. Explicit tasks may not be tapping into mentalization *per se* but could instead run into some communicational-pragmatic burden (Helming et al., 2014). The implicit ToM results have been swiftly questioned (Ruffman, 2014) on methodological grounds (Poulin-Dubois et al., 2018), or they were explained away, either entirely (Heyes, 2014), or by suggesting that infant mentalization is inferior to that of adults. It was suggested to be ape-like and not suitable for coordinating perspectives (Tomasello, 2018) or that it is perceptual and “low-level,” restricted to some sort of object tracking and physical perspective-taking system (Apperly and Butterfill, 2009; Low et al., 2016).

Nevertheless, a growing body of findings is proving to be increasingly difficult to explain without assuming adult-like meta-representational ToM in infancy. Observations that false beliefs can be ascribed to social partners without knowing their actual mental content (Kovács et al., 2021; Kampis and Kovács, 2022) suggest that infants attribute structured belief files (Kovács, 2016). Moreover, the ToM of 14-month-olds is capable of handling semantic representations that are in the appropriate “high-level” representational format for beliefs proper (Forgács et al., 2019, 2020). Based on these findings, it is well possible that ToM contributes to or enables language development rather than the other way around.

### 5.4 The social N400: is semantic processing mentalistic?

Recent findings on the so-called social N400 effect have profoundly challenged the received knowledge on the neurocognitive organization of language processing and its relation to social cognition (Rueschemeyer et al., 2015; Westley et al., 2017; Jouravlev et al., 2019; Hinchcliffe et al., 2020). When participants were required to track the comprehension of a confederate while reading semantically incongruous sentences together (“The boy had *gills*”), they exhibited an N400. Surprisingly, this occurred even when they heard context sentences beforehand (“In the boy’s dream, he could breathe under water”), which should have attenuated the N400 by providing interpretative context. The intriguing finding is that a social effect, which should have engaged pragmatic mechanisms, elicited a semantic response.

In a paradigm designed to directly manipulate the belief state of a communicative partner during language comprehension, even 14-month-olds produced a social N400 in response to the miscomprehension of a social partner (Forgács et al., 2019). In a puppet theater experiment, infants were presented with familiar objects that were always correctly labeled from their perspective but sometimes incorrectly labeled from the perspective of an observer. The observer, seated on the other side of the stage, had visual access to objects only when an occluder was lowered. First, an object was placed in front of infants (e.g., a cup), which was revealed to the observer as well; however, when the occluder moved back up, the observer turned away, and the first object was replaced by a second one (e.g., a car), unbeknownst to the observer. When the observer turned back, the second object was labeled (“car”), which was congruent for infants but incongruent with the false belief of the observer. Despite experiencing no semantic processing demands, infants produced an N400 (Forgács et al., 2019, 2020). Thus, the ERP indicator of language comprehension responded to a mentalistic manipulation, not simply a social one. These findings are relevant for ToM research because they demonstrate that false beliefs can be attributed as semantic content, which is compatible with a propositional meta-representational format. Conversely, ToM may be at full capacity already in infancy (Leslie, 1994). The findings are also remarkable from the perspective of experimental pragmatics because they show that not simply social cognition but specifically mentalization can impact language comprehension, not only at the level of pragmatic-inferential mechanisms but also at the level of semantic processing.

The social N400 appears to have two constituents: the *false* *belief N400* and the *social presence N400* (Forgács et al., 2022b). When presented with congruent and incongruent object-labeling events, adults showed an enhanced N400 response not only to incongruity but also to the mere presence of another person, in contrast to when they were alone. The typical N400 seems to be best explained as a semantic memory retrieval effort (Kutas and Federmeier, 2011; Brouwer et al., 2012; Urbach et al., 2020), which is evoked at all times but reduced when semantic predictions are met. Thus, the social presence effect can be understood as a lesser reduction of the N400 when someone is simply present. This may be due to a broader range of semantic elements remaining activated, which is likely to enhance potentially ensuing social interactions. The *false belief N400* can be elicited in adults as well, over and above the *social presence N400*. In the *false belief N400* paradigm, an observer is always present. However, an additional N400 effect is evoked only if participants are explicitly instructed to follow the comprehension of the other person (Forgács et al., 2022b)—just as in the information asymmetry social N400 experiments (Jouravlev et al., 2019). In sum, semantic processing seems to involve two mentalistic components: a spontaneous one, the *social presence N400*, and a strategic one apparent only following instructions, the *false belief N400*.

The *social presence N400* is evident already at 14 months of age, right at the developmental onset of the N400. Nonetheless, the effect appears only in response to incongruent labels, not to congruent ones (Forgács et al., submitted). It seems that infants ration their limited cognitive capacities to engage in semantic mentalization only when incongruent labels potentially incur divergent perspectives and false beliefs rather than when congruent labels require the attribution of true beliefs. In such cases, it may be sufficient to assume a shared, normative belief (Király et al., 2018). It may be argued that no attribution of beliefs is necessary for the social presence effect and that attributing perception may suffice. This may be true, but it is based on the assumption that the N400 is an indicator of perceptual processing. While it has been argued that the language system is fundamentally a reflex-like perceptual system (Fodor, 1983), the more broadly accepted view is that semantic mechanisms pertain to the conceptual system in some way. Additionally, the attribution of perception could also be viewed as a form of mentalization, as it involves ascribing an experience as a mental state.

The mentalistic social N400 is a riddle for pragmatic theories. For Neo-Griceans, social cognition, let alone mentalization, should not influence semantic mechanisms—only pragmatic inferences. For Post-Griceans, since inferential mechanisms may already be involved in developing explicatures, the impact of social cognition on semantic processing may not be unexpected. However, mentalization should be an input to the pragmatic module (together with the logical frame) and should not influence the initial lexical retrieval. The thought-provoking aspect of the mentalistic N400 is that none of these experiments were supposed to elicit an N400, as they did not pose any semantic processing demands *per se*. Instead, they well could have evoked ERPs associated either with ToM, including parietal or frontal responses (Liu et al., 2009; McCleery et al., 2011), or with pragmatics and contextual processing, such as the P600 (e.g., Van Berkum, 2009). Mentalization apparently impacted language comprehension not on a pragmatic but on a semantic level, which was not predicted by any pragmatic theories.

At a minimum, these results suggest that the ToM network may coordinate very closely with the language network (Paunov et al., 2019). The ToM network is a bilateral system, perhaps slightly more right-lateralized, with centers at the temporoparietal junction (TPJ) and the middle prefrontal cortex (mPFC) (Frith and Frith, 2003; Saxe and Kanwisher, 2003). It is part of a broader network of social cognition (Schurz et al., 2020). The language network is a more left-lateralized system of temporal and frontal regions (Binder et al., 1997). It has been argued that these two networks work independently (Shain et al., 2023), despite some apparent overlaps, and have stronger connections within themselves than between each other (Paunov et al., 2019). However, there are concerns with explaining the social N400 based on an interaction between the two networks. It is a lexical input that should trigger the ToM network (instead of the language processor), which in turn should activate the semantic system soon enough to produce an N400, yet not for semantic retrieval but to represent the mental state of a social partner. Thus, not only was an N400 not expected in the social N400 experiments (in the absence of semantic processing demands), and no other ERPs were observed (to indicate the activation of the ToM network), but specifically an ERP associated with the semantic system responded to the language comprehension and miscomprehension of a social partner.

It is true that belief attribution was accompanied by frontal effects in infants’ *false belief N400* experiments (Forgács et al., 2019, 2020). However, these effects were inconsistent between French and Hungarian infants, and frontal regions may be engaged during false belief processing for a variety of reasons beyond belief computations, from inhibitory control through response selection to resolving conflicting representations (Southgate, 2020). It is also true that the infant social presence study (Forgács et al., submitted) involved no false beliefs, only the tracking of another person’s experience of a semantic incongruity, which nevertheless still seems to qualify as at least some form of belief attribution. The overall pattern of results suggests that the semantic system is engaged in processing ToM in the mentalistic N400 experiments. Such an interpretation does not preclude the possibility that the ToM and language networks are separate systems that work closely together (Paunov et al., 2019; Shain et al., 2023; Fedorenko et al., 2024). The semantic system could work mentalistically without subserving other ToM functions.

## 6 Meaning as mentalization

**Figure 2:**
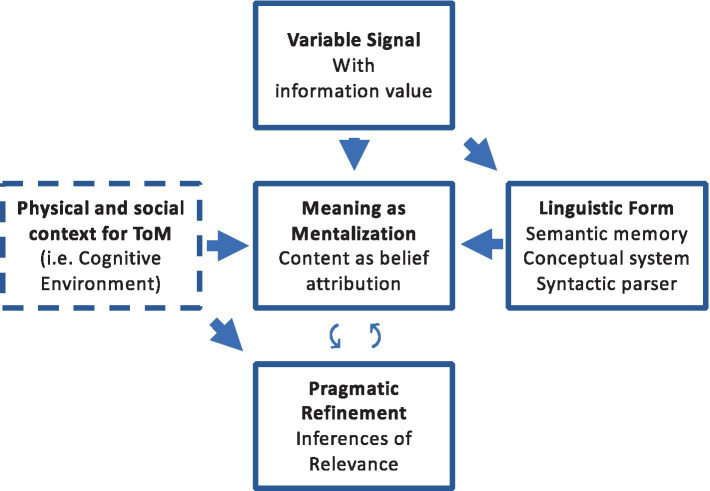
A novel model for establishing linguistic meaning through attributing mental content as intended meaning to social partners, based on the lexical input along with meta-communicative and other signals, the physical and social context, and the cognitive environment. Semantic content as a belief ascription may be updated based on logical and/or social inferential mechanisms during pragmatic enrichment. Mentalization may be optional for setting up communicative interactions and deriving pragmatic inferences. However, it seems indispensable for any theory involving (communicative) intentions that aims to explain meaning as the content conveyed in communication.

The main claim of this paper is that the semantic system may function in a mentalistic manner by storing, manipulating, and retrieving content based on belief attributions. The sensitivity of the N400 to mentalistic manipulations is not a curious detail but a functional characteristic of the semantic system. The idea is that the information transmitted via linguistic forms—the phonological-lexical input—triggers an unpacking mechanism of the belief the speaker intends to express based on semantic activations. Thus, interpreting utterances does not begin with merely looking up content in the database of the mental lexicon (as per the code model and the Neo-Griceans) or by generating a raw logical form that serves as an entry point for the mental encyclopedia (as per RT and the Post-Griceans). Instead, semantic content is the result of a memory retrieval of a likely intended sense based on the lexical evidence and the belief ascribed to the communicative partner as a probable piece of information (Figure 2).

Mentalization may or may not play a role in setting up communicative interactions by identifying communicative intentions (based on ostensive signals and/or engaging in joint attention) or in deriving pragmatic inferences (of social cognition and/or logical reasoning). However, it may be crucial exactly in between the two, when meaning is arrived at—where intentions may matter the most. The content linked to linguistic forms during language acquisition, as well as during everyday language comprehension, may be viewed as an attribution within the constraints of both the code-like features of language and the social cognitive dimensions of human communication. The structural properties of language and word forms may help limit the scope of the mentalistic attributions of intended content, while pragmatic inferences may help further specify and adjust it, if necessary. In contrast to Vygotsky’s and Bruner’s studies, where scaffolding by the social world fills the minds of children from the outside (Wood et al., 1976), the present approach proposes the reverse direction. The social aspect may work from the inside out in the form of social cognition, from the minds of children toward the minds of social partners to acquire meaning by attributing beliefs. Thus, semantic content may not be identified in the external world, as referents discovered during social interactions, but in the internal worlds of communicators, as hearers’ best guesses for belief ascription.

The recognition of communicative intentions, in the sense of *intention to communicate*, maybe the entry point for ascribing beliefs to social partners. The mentalistic attribution of potential content may be the richer the more complex the code is, such that pointing is superseded by pantomiming, which is superseded by language proper, be it whistle, sign, or verbal language use. The recognition of communicative intentions may not simply aid reference resolution via attention guidance, after which relevant information can be transmitted regarding the world (of objects, actions, or properties). Instead, it may initiate the attribution of what the other person may have in mind (a particular object, action, or property). By the time linguistic information transmission commences, the referent of a spoken word may not be identified as a physical object but as the mental representation of the object attributed to the communicative partner.

It may be argued that no mentalization is required once joint attention or ostensive cues have done their job because infants may simply take the attended object to be the referent of the word to establish word-to-world mappings. They need no representation of the mental content of the communicative partner by the time information is transmitted. However, the content of the word would still be enormously difficult to determine based solely on the tracking of attention, goals, and physical objects, as highlighted by the “gavagai problem” (Quine, 1960). Markman’s constraints may provide some aid on a pragmatic level, but they do not seem to solve the matter comprehensively, especially at the very early stages of world learning. The problem largely evaporates if one assumes that the referents we interact with, communicate, and talk about are not simply in the physical world but inside the minds of speakers, in contrast to traditional views on language acquisition.

The transmission of the signal may be exploited to narrow the range of possible mentalistic attributions, which specify communicative intentions, now in the sense of *meaning as intended*. The informative intention could thus be viewed as the particular attributed belief. Such a mechanism could account for both the social acquisition of linguistic forms (from words to grammar) and the interpretational wiggle room language always seems to leave. Pragmatic inferences may further narrow the remaining ambiguity but may not necessarily involve mentalization. Social-contextual adjustments may be made optionally based on information available in the cognitive environment and/or the common ground, and sometimes updating the initial content attribution may be unavoidable, but not always.

The semantic system would still accumulate, store, and utilize statistical, taxonomic, and other structural regularities of the incoming signal to provide a better springboard for its main function of attributing meaning. As noted by Bruner (1990), linguistic meaning does not seem to be looked up from a data table but is rather reinvented from incoming raw materials in a creative process of “meaning making.” Viewing the semantic system as a mentalistic system could bridge the gaps between word, sentential, and contextual meaning by treating them as the same kind of belief attribution by the language system, albeit with gradually increasing complexity. We may rely more on the code in particular routine situations, from formulaic language to other conventions, as proposed and perhaps overgeneralized by the Speech Act theorists (Austin, 1962; Searle, 1979) or Millikan’s (2005) direct perception model. However, even such interpretative best guesses could be mentalistic in nature and not qualitatively different from semantic ToM efforts when communication does not unfold as predicted.

This view could account for the mentalistic social N400 findings without appealing to a peculiar interaction between the language and the ToM networks. The idea is that the language system was not recruited by the ToM network but worked independently, carrying out mentalistic functions. The current proposal argues that these experiments were not revealing exceptions but rather the modus operandi of the semantic system. The findings of Fedorenko and colleagues that classic ToM and language tasks do not engage the other network do not refute the idea that the language network may function mentalistically.

The finding that adults produce a false belief social N400 only when explicitly requested to do so (Forgács et al., 2022b), while infants show it spontaneously (Forgács et al., 2019), suggests that language learners may rely more heavily on belief attributions to identify intended meanings than adults. With accumulating conversational routine, adults may be less prone to invest additional neural resources in strategic mentalization beyond spontaneous mentalization. During language acquisition, the semantic system may be optimized toward a generic model of an idealized speaker. With the gradual expansion of lexical databases, linguistic conventions, and conversational routine, semantic mentalization may increasingly resort to normative attributions to a default speaker. By adulthood, only when interactions and conversations take unexpected turns may personalized mentalization retake the lead.

A possible objection to the semantic system always functioning mentalistically is that it would imply no difference between social and non-social language input. The present framework proposes that the amplitude of the N400, being a graded ERP, reflects varying neural processing demands not only in response to lexical retrieval but also to mentalization. The various technological innovations that allow linguistic input to be provided without a speaker actually being present in person (from writing systems to audio recordings) may hack into the proper cognitive domain of the semantic system. Classical psycholinguistic experiments testing individuals alone may have tapped into a special case of language processing based on generic semantic attributions to a default speaker. When linguistic stimuli are encountered in the physical presence of a social partner, additional semantic attributions are spontaneously generated for the specific individual beyond the generic model. The system’s functioning is further geared up when the other person experiences a false belief, and the conversation may be derailed. Attributions of meaning may be simpler if the interlocutors are closer to each other’s idealized default speaker model. In a close language community, each individual’s idealized speaker model is based on a highly similar body of language input, toward which the code structures are statistically optimized. The statistical structures of the semantic memory system that psycholinguistic experiments have described in great detail may reflect these statistical features, but the proper function of the system may still be determining what was meant by communicative partners.

It may also be argued that the social N400 is a result of social facilitation. While such an explanation may be possible for adults’ social presence effect, it does not work for infants’ because it appeared only in a semantically incongruent condition, which suggests its strategic employment. This explanation also cannot account for the *false belief N400* effect because it appeared in adults only after explicit instructions, indicating again a strategic element. Of course, future studies are necessary to further scrutinize and gather additional evidence in support of the theory.

## 7 Conclusion

Can meaning be understood as unintended? Is meaning an abstraction in the world or a psychological phenomenon in the mind? It seems paradoxical to argue that intentions, especially when they are communicative, are not attributed to social partners. One may reverse the question: how much of establishing intended meaning is *not* mentalistic? The present study proposes that, instead of relying on decoding and pragmatic mechanisms, meaning is directly interpreted as it is intended. Meaning may be the information mentalistically attributed as a belief to a communicative partner.

## Author contributions

BF: Writing – original draft, Writing – review & editing.
